# Infrequent case of leucoencephalopathy after electric shock trauma

**DOI:** 10.1016/j.radcr.2025.02.066

**Published:** 2025-03-18

**Authors:** Julián Eduardo Grisales, Valentina Quintana, Maria Fernanda Laverde, Camilo Hernán Bonilla, Juan Camilo Márquez, Alberto Masaru Shinchi

**Affiliations:** a School of Health Sciences, Universidad Icesi, Cali, Colombia; b Department of Radiology and Diagnostic Imaging, Fundación Valle del Lili, Cali, Colombia; c Department of Neurology, Fundación Valle del Lili, Cali, Colombia

**Keywords:** Electric injury, Electric shock, Encephalopathy, White matter, Hypoxic ischemic

## Abstract

Due to the low frequency and nature of electric injuries, and the different implied mechanisms of trauma, which can manifest in different organs and systems depending on the time of exposure, voltage, amperage and intrinsic resistance of body tissue, the presentation and consequences of the trauma are heterogeneous, and difficult to characterize.

We present the case of a previously healthy adult patient who had a high voltage electric injury, resulting in a diffuse brain leukoencephalopathy with delayed image presentation in MRI as an early complication, and a short review of the mechanisms of electric injury which will help us to understand this pathology and its manifestations in the central nervous system.

## Introduction

Electric discharge trauma (EDT) is an infrequent entity, often encountered in young male adults with Laboral exposure. EDT can affect multiple organs and systems, frequently producing local, cardiovascular and neurological injury [1]. Neurological complications reported [2] include compromise of central nervous system, peripheral nervous system and even the neuromuscular junction, having diverse presentations such as seizure, intracranial hemorrhage, stroke, hypoxic-ischemic encephalopathy, transverse myelitis and peripheral neuropathy. These complications can appear in the patient with an early or delayed onset after the trauma, and may have variable duration, from temporary to definitive. Most case reports related with neurological EDT complications were diagnosed long time after the moment of the injury [2,3]; which in our case, could be associated in the same episode due to the early onset of symptoms and neuroimaging changes.

## Case presentation

**Fig. 1:**
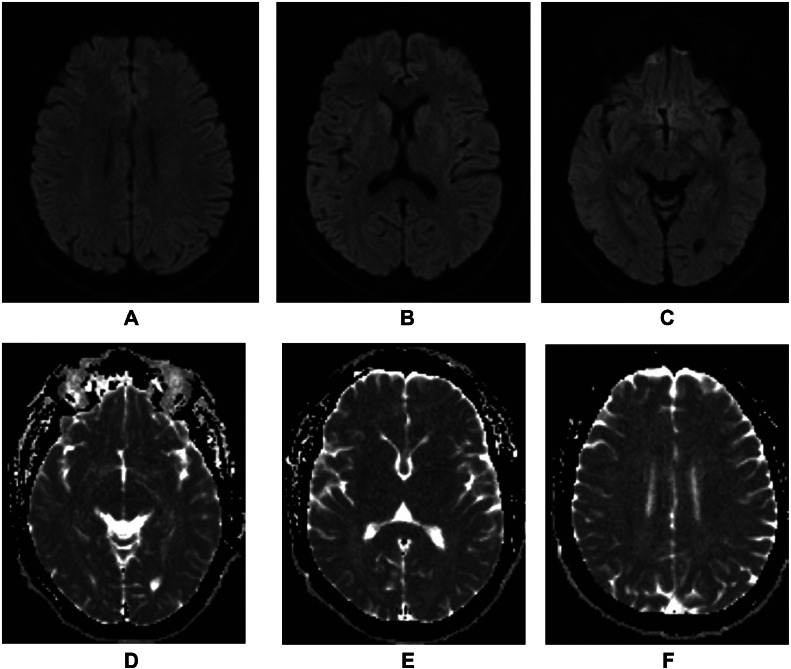
Diffusion weighted imaging, being B1000 (A, B, C) and ADC (D, E, F) without evidence of injuries.

**Fig. 2:**
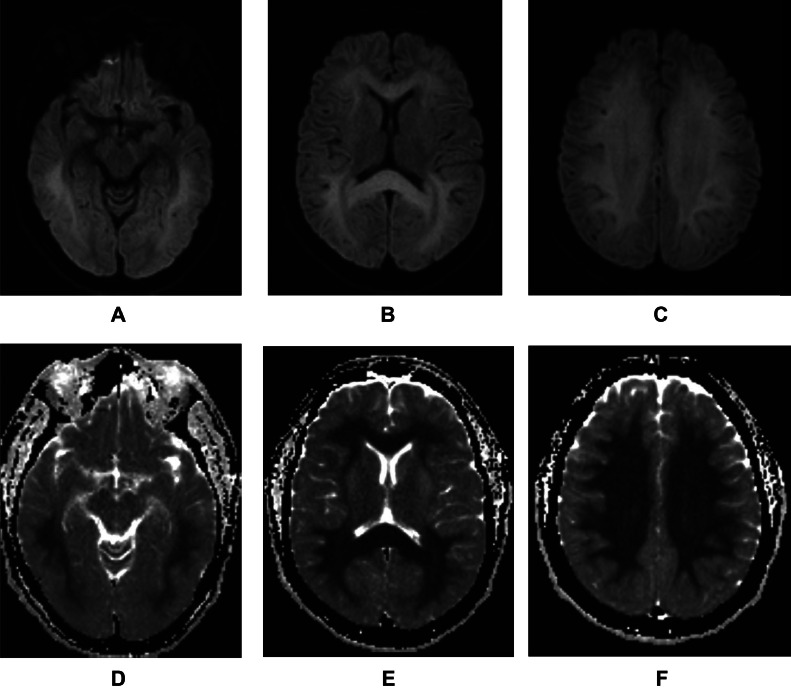
Diffusion weighted images 9 days after the event. Notice the diffuse increase in the signal intensity of the white matter in B1000 sequences (A, B, C) matching the decrease in the signal intensity in ADC, consistent with restricted diffusion. (D, E, F).

We present the case of a 37 year old male patient, working for an electricity company, who was previously healthy and suffered an electric burn injury after direct contact with a high voltage cable and his right hand. He had 2 episodes of ventricular fibrillation with return to spontaneous circulation after high quality pulmonary resuscitation. Afterwards, he coursed with multiple early epileptic crises, treated with anticonvulsants, which was followed by a diagnostic brain magnetic resonance (BMR), showing no abnormalities at the time given (Fig. 1). The patient received 72 hours of therapeutic hypothermia protocol. Nine days after the initial event, the patient only performed periodical eye opening without contact with the environment. He had flaccid tetraplegia with preservation of brainstem reflexes. In this moment, it was decided to perform a new BMR which showed diffuse and symmetric restricted diffusion of the subcortical, periventricular white matter, involving the corpus callosum and Putamina, with subtle representation in FLAIR sequences; these findings were associated with narrowing of the sulci and subarachnoid spaces (Fig 2).

The patient required a long stance in the critical care facility, requiring intubation, tracheostomy and gastrostomy. At discharge the patient performed ocular opening but without interaction with the environment.

## Discussion

We present the first case of bilateral and symmetric extensive restricted diffusion of white matter after a direct, high voltage EDT. Restricted diffusion of white matter after electric shock can be explained with the physical properties of the axons, which are conductive, producing retrograde energy flow through the vast interconnection of neurons, producing symmetrical injuries despite the way of access through the right hand [4]. Energy then transmits to high resistance areas, such as the neuronal bodies and junctions, producing respective injuries to the tissue.

The effects of EDT are variable, depending on the different properties of conductivity and resistance of the tissues involved, which follows multiple types of injury, being direct (burns) and indirect (hypoxic ischemic encephalopathy due to cardiac arrest and arrhythmia or head trauma for falling after loss of consciousness) [5]. There is a third implied mechanism which is called electroporation, which consist in an irreversible change in the tertiary configuration of proteins in cell membrane, altering transmembrane equilibrium of ions, with the consequent failure of Sodium-potassium ATPase pump, altering production of energy and cellular function, followed by cellular death.

We consider electroporation as the physiopathological mechanism implied in our patient, given the extensive and symmetric restricted diffusion of subcortical white matter. The patient also presented 2 episodes of ventricular fibrillation, reverted through defibrillation, which could condition hypoxic-ischemic encephalopathy associated. Nevertheless, the restricted diffusion of our patient spared the gray matter of the cortex and basal ganglia, which differentiates of the usual presentation of hypoxic-ischemic encephalopathy, that involves mainly these structures, which are metabolically more demanding [6].

There is another classification of the electric injury as immediate or permanent using the Cherinton classification: Gliosis, neuronal cromatholysis in pathology, hemorrhagic focuses and brain infarcts; the extension and severity of the injuries depends mostly of the time and strength of the electric flow.

The electromechanic commission [3,4] defines high voltage as a discharge superior to 1000 volts, which usually generates burns in the skin which had contact with the source. Cherinton classification divides it in 4 stages:

1. Immediate and transitory: Symptoms that present immediately after the event, such as confusion, altered state of consciousness which resolves in minutes or hours. There can be hyperintensities in T2 weighted sequences or diffusion, which can resolve partially or totally in a posterior control.
2. Immediate and permanent: gliosis, neuronal chromatolysis in pathology, hemorrhagic focuses and brain infarcts. Depends mostly on the voltage and time of exposure.
3. Delayed and progressive: the clinical manifestations are more insidious, usually with not proportionate findings in imaging, such as myelopathy, white substance involvement, basal ganglia involvement and disruption of the main motor tracts which can result in permanent disability and bad prognosis.
4. Associated to the event: For example, the post traumatic hematomas produced during falls or hypoxic-ischemic encephalopathy secondary to cardiac arrest.

## Conclusions

Electric shock injury has a wide spectrum of manifestations, which depend on the transference of energy to the different tissues, according to their conductance or resistance. In some occasions, it is not possible to determine the stigmata of EDT evidently during the clinical examination, making the radiological evaluation a crucial step in the workup of these patients. [7]

In the central nervous system, we usually observe changes in the signal intensity of brain parenchyma which can be reversible or permanent, depending on the time of exposure and voltage, which are variables that should be included in the clinical record to orientate management. [8] Imaging patterns can help us determine the possible mechanism of the injury of the neuronal structures implied, having in mind the distribution pattern, evidence in diffusion sequences and T2 weighted sequences and follow up imaging and clinical development, with the consequent impact in decision making for management, prognosis and rehabilitation of patients.

## Patient consent

The reported case was reviewed and approved, and individual patient consent was obtained following institutional guidelines.
